# A global database to catalogue the impacts of agricultural management practices on terrestrial biodiversity

**DOI:** 10.1016/j.dib.2023.109555

**Published:** 2023-09-09

**Authors:** Jonathan Bonfanti, Joseph Langridge, Damien Beillouin

**Affiliations:** a CIRAD, UPR HortSys, F-34398 Montpellier, France; b HortSys, Univ Montpellier, CIRAD, Montpellier, France; c FRB-CESAB, Fondation pour la Recherche sur la Biodiversité – Centre de Synthèse et d'Analyse sur la Biodiversité, 5 rue de l'école de médecine 34000 Montpellier, France; d Cirad, UPR HortSys, F-97285, Le Lamentin, Martinique, France

**Keywords:** Agriculture, Land-management practices, Meta-analysis, Taxonomic groups, Richness, Abundance, Biomass

## Abstract

Habitat loss and degradation due to global agriculture land use is a major threat to biodiversity. Identifying agricultural management practices that mitigate these impacts is urgently needed. Thousands of experiments have been conducted worldwide in the last decades to compare the impacts of various agricultural management practices on biodiversity. The magnitudes of difference in biodiversity responses between pairs of agricultural practices, i.e. effect sizes, have now been synthesised in a growing number of meta-analyses. Yet, each meta-analysis generally focuses on a specific type of farming practice and on specific taxonomic groups, or a single region. Meta-analyses could furthermore yield different or sometimes opposite results for the similar research questions. Gathering all the effect sizes in one single dataset helps to critically assess and weigh the available evidence across all studied practices, taxonomic groups and geographical areas, and provide stakeholders a solid base to better inform their decisions. Here, we present a comprehensive dataset of 200 published meta-analyses gathering 1885 effect sizes based on more than 14 000 primary studies. We detail the effect of 8 main individual field practices (e.g. pest and disease management, amendment and fertilisation), 3 agricultural systems (e.g. organic farming, conservation agriculture) and 2 landscape level interventions (i.e. landscape complexity, land-use change). Our dataset covers numerous taxonomic groups over 14 phyla, including animals (e.g. birds, insects), microorganisms (e.g. fungi, bacteria), plants (e.g. trees, weeds). The dataset presented provides a resource to support decision-makers, farmers, and conservation ecologists alike for managing agricultural land for biodiversity.

Specifications Table

**Table:** 

Subject	Biological sciences: **Biodiversity** (or Environmental science: Ecology)
Specific subject area	Terrestrial biodiversity, land management practices in croplands
Type of data	Six tables: - **Table “Header names”**: in rows, lists the header names of the tables “included studies”, “excluded studies” and “effect sizes and qualitative data”; in columns, gives a description of each item and its filling procedure. **- Table “Included studies”**: in rows, the list of 200 included meta-analysis after screening steps; in 7 columns their metadata (e.g. ID, title, date). **- Table “Excluded studies”**: in rows, the list of 3953 excluded literature references (resulting from the screening steps); in 7 columns their metadata and a reason for exclusion. **- Table “Effect sizes and qualitative data”**: in rows, the 1885 effect sizes extracted from the 200 included meta-analyses; in columns, 36 characteristics of the effect sizes (e.g. Intervention type, Biodiversity group, Vote-counting effect…). - **Table “Glossary & coding”**: lists the entries in the table “Effect sizes and qualitative data” for the columns related to the Intervention and mode of comparison, the biodiversity Outcome and the metrics of the effect sizes, and gives a definition for each item when appropriate. **- Table “Test-list”**: in 39 rows, lists the meta-analyses used to make a preliminary test of the performance of the literature search; in 5 columns their metadata.
How the data were acquired	Following a systematic review procedure, we performed a search in four scientific databases to retrieve all relevant peer-reviewed meta-analyses quantifying the effects of agricultural management practices in croplands on biodiversity. We obtained a total of 6709 references from which 2555 duplicates were removed. Two screening steps were carried out: (i) title and abstract, to exclude articles that clearly failed to meet our inclusion criteria, (ii) full-text of all remaining articles to further exclude non-relevant articles. Article eligibility was based on pre-defined inclusion and exclusion criteria reflecting the objectives of our study. Finally, within each retained meta-analysis, we identified the effect sizes that present the effects of an agricultural management practice on biodiversity and we manually extracted their related qualitative data (e.g., Intervention, Comparator, Moderator(s), Biodiversity Outcomes).
Data format	Raw
Description of data collection	After the two-steps screening process, 200 meta-analyses met our inclusion criteria and were retained for subsequent analysis. Data were extracted and archived in an Excel spreadsheet. The selection process was undertaken by two curators, and the consistency of their decision was assessed with a Kappa test. The retained meta-analyses were characterised by their bibliographic metadata (e.g. publication title, authors, etc.). The effect of agricultural management practices on biodiversity were characterised by: (1) the type of intervention (e.g. individual practices such as fertilization or tillage, agricultural systems such as organic agriculture, etc.) and their comparators (e.g. natural habitat, conventional agriculture, etc.), (2) the included study characteristics or effect moderators (e.g. climatic conditions, soil characteristics, etc.), (3) the characteristics of the population (taxon) and the outcomes, and (4) the details on the effect size (e.g. type of metrics, number of paired data, etc.).
(*continued on next page*)

**Table:** 

Data source location	Raw data from meta-analyses: the list of all retrieved references from searches performed is provided. All data extracted from retained articles in accordance with our systematic review protocol are provided in Excel spreadsheets. Dataset (Excel spreadsheets) are hosted on the online repository “CIRAD Dataverse” by research unit CIRAD, UPR HortSys, F-34398 Montpellier, France.
Data accessibility	Repository name: CIRAD Dataverse Data identification number: Bonfanti, Jonathan; Beillouin, Damien, 2023, “A global database to quantify the impacts of agricultural management practices on terrestrial biodiversity,” https://doi.org/10.18167/DVN1/RIRTOT, CIRAD Dataverse, V3, UNF:6:UOGjnVxAQPIZxRTMtaOV3w== [fileUNF] Direct URL to data: https://doi.org/10.18167/DVN1/RIRTOT

## 1 Value of the Data

(1) We provide an up-to-date standardised catalogue of existing meta-analyses evaluating the effects of all agricultural management practices on biodiversity.
(2) The database contains effect size qualitative data providing information on the impacts of a large range of farming practices on terrestrial biodiversity across the globe.
(3) The database can be used to build an evidence map of the effects of various agricultural management practices on terrestrial biodiversity in croplands, highlighting knowledge gaps and knowledge clusters in terms of available evidence syntheses.
(4) The structure of the different tables included in our databases facilitates the inclusion of the results of future relevant meta-analyses.
(5) The structure of our database also allows for inclusion of quantitative data for each effect size, which may be analysed in a second-order analysis.

## 2 Objective

The objective of this study was to collect and describe the available evidence in terms of meta-analyses dealing with the impacts of agricultural management practices on terrestrial biodiversity at the global scale. We present the precise methodology used to conduct the review and extract qualitative data, following recent guidelines and standards in order to increase transparency, reproducibility and FAIR principles. Our rigorous reporting methods and dataset structure should allow for the process of updating i.e. including new references (rows) and/or new descriptors of each reference (columns).

## 3 Data Description

The dataset consists of one Excel file composed of 6 tables (i.e. sheets). Each table is described hereafter.

### 3.1 Table “Header names”

This table lists the header names of the tables “Included studies”, “Excluded studies” and “effect sizes and qualitative data”. To ease the understanding of the columns contained in these tables, we provide a brief description of each header name and the type of value contained in each column.

### 3.2 Table “Included studies”

This table lists the meta-analyses included in our systematic review, i.e. that were included after the screening procedure described hereafter and thus met our inclusion criteria (see notably §4.3). This table consists of 201 rows x 7 columns. Each row represents one included meta-analysis, the 7 columns describe the metadata of each study. The metadata are: unique ID, title, abstract, journal of publication, authors’ names, DOI, year of publication.

**Fig. 1:**
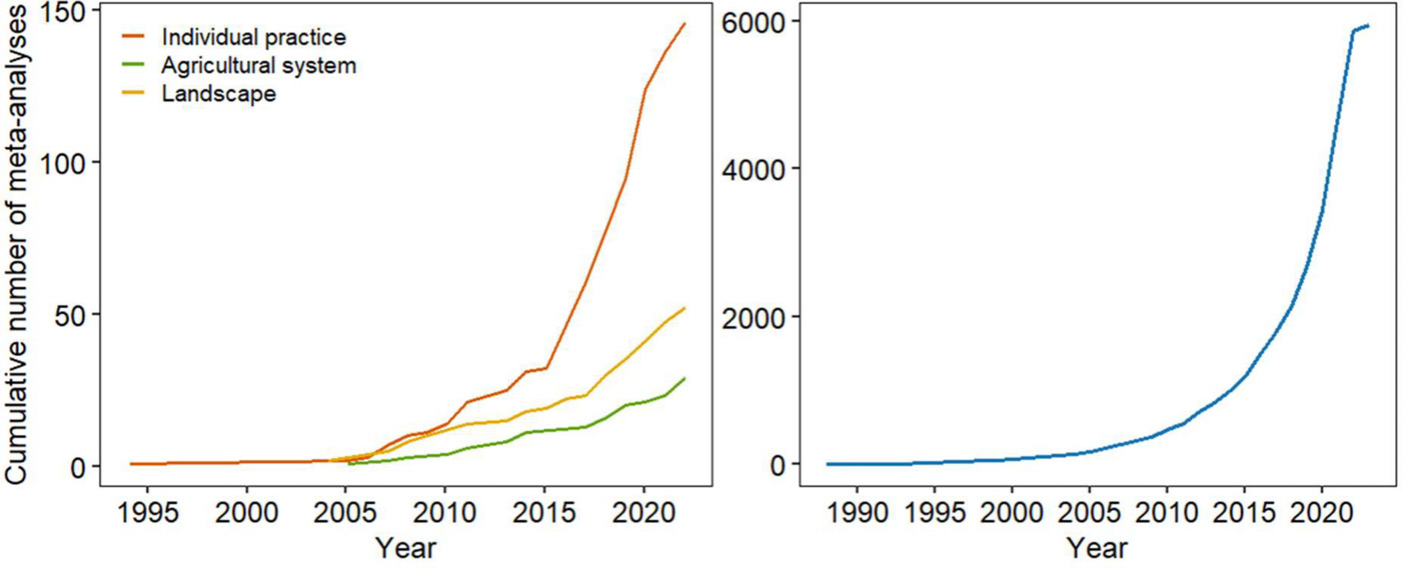
Cumulative number of meta-analyses included in our database (left panel) showing the effects of agricultural management practices on biodiversity over time, compared to the cumulative number of meta-analyses referenced in the Web of Science over the same period (right panel). Left panel: One meta-analysis may be attributed to one or several types of intervention, depending on the effect size(s) that were extracted from it. We considered 3 types of intervention: ‘Individual practice’ (in orange) for individual management practices (e.g. tillage, use of biocide…), ‘Agricultural system’ (in green) for sets of practices tested together (e.g. organic agriculture) and ‘Landscape’ for landscape-scale management studies (land-use change, landscape complexity). Right panel: We extracted from the Web of Science (in January 2023) the number of references containing “meta-analysis” in all fields, belonging to ‘Environmental sciences’ categories only, which represents 5938 results.

We illustrate the publication through time of relevant studies for our systematic review in Fig. 1.

### 3.3 Table “Excluded studies”

This table lists the studies that were excluded from our systematic review, i.e. that did not meet our inclusion criteria. This table consists of 3954 rows x 7 columns. Each row represents one excluded study, the 7 columns describe the metadata of each study and a rationale for exclusion. The metadata are: unique ID, title, abstract, journal of publication, authors’ names, DOI, year of publication. The rationale for exclusion is qualified using 6 different reasons for exclusion that may concern: accessibility, absence of biodiversity outcome, irrelevant context, absence of relevant intervention, out of scope, other reasons.

### 3.4 Table “Effect sizes and qualitative data”

**Fig. 2:**
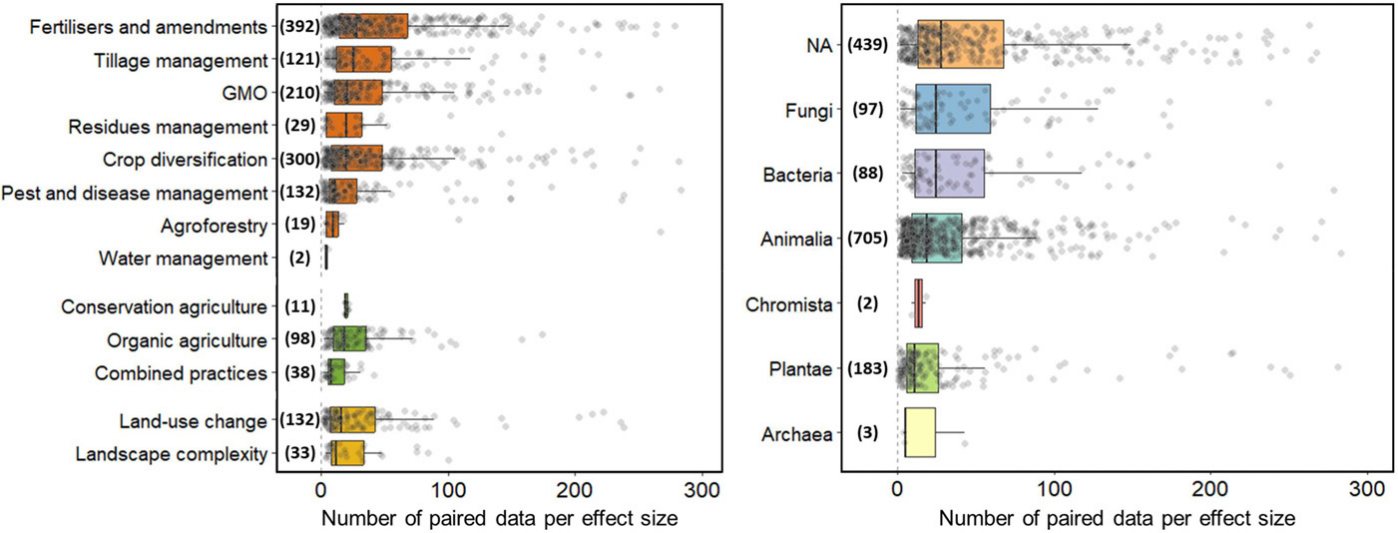
Total number of effect sizes (in brackets) and mean number of paired data per effect size (box plots, points) for each agricultural management practice (left panel) and taxonomic kingdom (right panel). x-axis: number of paired data used to calculate each effect size, presented in boxplots with jitter points (for clarity, in both panels 28 jitter points in total were hidden as they represent a number between 300 and 800); in orange: individual practices, in green: agricultural systems, in yellow: landscape scale management. y-axis: agricultural management practices (left) and kingdom (right), with in brackets the number of effect sizes. A total of 368 effect sizes are not represented as they do not provide the number of paired data used for their calculation. GMO: genetically modified organism; NA: kingdom not precised or multiple kingdoms are involved within the effect size.

This table lists the description of the effect sizes (i.e. an aggregated mean effect resulting from the comparison of an agricultural management and a control, expressed with a biodiversity metric) that were available within each included meta-analysis. One meta-analysis may contain several effect sizes. This table consists of 1886 rows x 36 columns. Each row represents one effect size. It is notable that each effect size was originally calculated from a specific number of paired data which is usually given by the authors of each meta-analysis. We present the total number of effect sizes and paired data for each of the 13 agricultural land management practices and for taxonomic kingdoms in Fig. 2.

### 3.5 Table “Glossary & coding”

This table lists the typology of intervention, comparisons and biodiversity (taxa and metrics) used. Thus, it details the entries given in the columns ‘Inter_type’, ‘Inter_R1’, ‘Scenario_comp’, ‘Kingdom’, ‘Phylum’, ‘Class’, ‘Order’, ‘ES_metric_output_R1’ in the table “Effect sizes and qualitative data”. To ease the understanding of these columns, we provide a brief definition of each entry.

Example: The different modalities of intervention are shown in the column ‘Inter_R1’ that may be filled with e.g. *Crop diversification, Agroforestry, Landscape complexity*, etc. Each term is thus defined, such as e.g. *Agroforestry: A practice containing at least two plant species in interaction whose at least one is a woody perennial and at least one is managed for crop production or forage; includes e.g. alley cropping with trees, shade monoculture.*

### 3.6 Table “Test-list”

This table lists the ‘test-list’ used to test the performance of our search strings (see §4.2.3). It consists of a list of 39 meta-analyses that we considered relevant for our present systematic review before searching through online databases.

## 4 Experimental Design, Materials and Methods

This methodology consists of a review protocol that follows a framework for synthesising evidence presented in meta-analyses. To ensure replicability, transparency and objectivity, the procedures broadly follow the standards and guidelines of the Collaboration for Environmental Evidence (Environmental Evidence journal ‘systematic review protocol’ accessed in June 2021; [1]). Our methods also follow ecology-focused guidelines [2], as well as closely-related examples of existing meta-analyses [3,4].

### 4.1 Research questions and PICO-C components

Our aim is to gather meta-analyses on the following question: what are the impacts of agricultural management practices on terrestrial biodiversity?

More precisely, we aim to analyse:

1. The diversity of agricultural management practices investigated in the existing meta-analyses (e.g. tillage, organic agriculture, landscape complexity…);
2. The range of taxonomic groups studied (e.g. bacteria, earthworms, birds…), and/or functional groups (e.g. weeds);
3. The different biodiversity metrics studied (e.g. species richness, abundance, diversity indices…).

From this primary research question, we detail the following components following a PICO-C framework [5] (Table 1). No date restriction is applied. All climatic zones are considered.

**Table 1:** Components of the review questions.

PICO-C component		Definition
Population:	All terrestrial and semi-aquatic taxa	This included microorganisms, vascular and nonvascular plants, invertebrates, and vertebrates.
Intervention	All agricultural management practices	Any individual practice (e.g. tillage, use of biocide, amendments…), set of practices or agricultural system (e.g. organic agriculture) studied at field or farm scale, or landscape metric in croplands.
Comparator	Two types of comparators: (i) Different intensities of agricultural management; (ii) Natural habitat	Concerning individual agricultural practices and for the agricultural system scale, we used the more intensified management as the control. We defined intensification as either i) an increase in the use of the external input (e.g. chemical, N fertilizer, mechanisation), or ii) a decrease in the number or evenness of cultivated plant over space or time, or iii) at a landscape scale: a decrease of landscape complexity or a change in land use.
Outcomes	All biodiversity metrics	Effect sizes, i.e. a weighted mean comparison between two modalities of agricultural management practices, usually an Intervention (treatment) and a Comparator (control). The effect size can average different biodiversity metrics (e.g. species richness, abundance, diversity indices…). It can be expressed in different effect size metrics (e.g. ratio, Hedge's g…) completed with indicators of precision (e.g. confidence interval).
Context	Type of publication	Only first order meta-analyses conducted anywhere in the world in croplands or agricultural contexts, with no temporal restriction. We did not consider vote-counting studies as meta-analyses.

### 4.2 Searching for relevant meta-analyses

We conducted the search in four online databases using the refined search string on 21st July 2021 and updated the results of our literature search in September 2022 (restricting our research to articles published later than August 2021). We obtained a cumulated number of 6682 records.

#### 4.2.1 Languages

Only English terms were included in the searches and, from the returned articles, those in either English and French were assessed and read, due to limited resources and the languages understood by the review team.

#### 4.2.2 Search string(s)

The search string was built during a scoping exercise in Web of Science (see §4.2.3) for use in publication databases, we present it in Table 2.

**Table 2:** Search strings used to identify relevant meta-analyses in four research engines. Wildcards: The asterisk (*) represents any group of characters, including no character, the dollar sign ($) represents a single character or no character, the quotation marks (“ “) searches for an exact phrase. We inserted line breaks and bold characters for operators to ease the reading.

Search engine	Search string
WOS, Scopus*	(meta-analysis OR “systematic review” OR meta-regression OR “quantitative synthesis” OR “global synthesis” OR metaanalysis OR “quantitative review”) **AND** (crop* OR agricultur* OR farm* OR land-use OR landscape OR agroecosystem$ OR “nitrogen addition” OR “N add*”) **AND** (system$ OR practice$ OR management OR conventional OR “alternative agriculture” OR “alternative farming” OR organic OR agroecolog* OR “conservation agriculture” OR biodynam* OR permaculture OR IPM OR “integrated pest management” OR low-input OR “embedded natural” OR agroforestry OR biocontrol OR “urban agriculture” OR till* OR fertiliz* OR amendment$ OR manure OR biocide$ OR pesticide$ OR fongicide$ OR herbicide$ OR abandonment OR set-aside OR fallow$ OR “mixed crop-livestock$” OR “integrated crop-livestock$” OR “diversified crop-livestock$” OR “vegetation strip$” OR “insect strip$” OR “flower strip$” OR intensification OR diversification OR rotation OR *inter-crop* OR cover-crop* OR mixture OR “crop sequence$” OR polyculture OR semi-natural OR irrigation OR biofuel OR energy-crop$ OR simplification OR grassland$ OR “farm size$”) **AND** (biodiversity OR diversity OR richness OR abundance$ OR evenness OR divergence OR dispersion OR structure OR function OR index OR migration OR extinction OR coloni$ation OR communit* OR assemblage$ OR species OR population* OR “soil fauna” OR “soil diversity” OR “soil biota” OR “soil organism$” OR “soil biology” OR bacteria$ OR fung* OR mycorrhiza$ OR microb* OR arthropod* OR insect$ OR collembol* OR arachnid$ OR spider$ OR myriapod$ OR mollusc$ OR gasteropod$ OR annelid$ OR earthworm$ OR reptile$ OR amphibian$ OR avifauna OR bird$ OR mammal$ OR *fauna OR weed$ OR plant$ OR pollinator$ OR decomposer$ OR “ecosystem engineer$” OR pest$ OR disease$ OR “natural ennem*” OR “microb* regulator$”)
Ovid	((meta-analysis or “systematic review” or meta-regression or “quantitative synthesis” or “global synthesis” or metaanalysis or “quantitative review”) AND (crop* or agricultur* or farm* or land-use or landscape or agroecosystem$ or “nitrogen addition” or “N add*”) AND (system$ or practice$ or management or conventional or “alternative agriculture” or “alternative farming” or organic or agroecolog* or “conservation agriculture” or biodynam* or permaculture or IPM or “integrated pest management” or low-input or “embedded natural” or agroforestry or biocontrol or “urban agriculture” or till* or fertiliz* or amendment$ or manure or biocide$ or pesticide$ or fongicide$ or herbicide$ or abandonment or set-aside or fallow$ or “mixed crop-livestock$” or “integrated crop-livestock$” or “diversified crop-livestock$” or “vegetation strip$” or “insect strip$” or “flower strip$” or intensification or diversification or rotation or inter-crop* or cover-crop* or mixture or “crop sequence$” or polyculture or semi-natural or irrigation or biofuel or energy-crop$ or simplification or grassland$ or “farm size$”) AND (biodiversity or diversity or richness or abundance$ or evenness or divergence or dispersion or structure or function or index or migration or extinction or coloni$ation or communit* or assemblage$ or species or population* or “soil fauna” or “soil diversity” or “soil biota” or “soil organism$” or “soil biology” or bacteria$ or fung* or mycorrhiza$ or microb* or arthropod* or insect$ or collembol* or arachnid$ or spider$ or myriapod$ or mollusc$ or gasteropod$ or annelid$ or earthworm$ or reptile$ or amphibian$ or avifauna or bird$ or mammal$ or fauna or weed$ or plant$ or pollinator$ or decomposer$ or “ecosystem engineer$” or pest$ or disease$ or “natural ennem*” or regulator$)).ab.
Google Scholar⁎⁎	(meta analysis OR “quantitative review”) AND (crop* OR agr* OR land*) AND (system OR practice OR fertil* OR intensi* OR till* OR ecol* OR complex* OR amend* OR diversifi*) AND (biodiversity OR species OR population* OR communit* OR biota OR richness)

⁎ field tag used in WOS was TS=(), field tag used in Scopus was TITLE-ABS-KEY ()

⁎⁎ google scholar only allows up to 256 characters, thus a simplified search string was used (cf. Haddaway 2015)

*Search terms, languages and restrictions*. Sensitivity is favoured over specificity. Sensitivity implies that the emphasis of the search procedure is put on collecting the largest selection of relevant articles at the risk of also obtaining a high number of non-relevant articles (hence increasing the duration of the screening steps). We thus firstly built a relevant keywords list - reflecting the PICO-C components - that we then used for building an initial search string list.

*Main differences between search strings and refining.* We initially wrote a search string to question WoS and Scopus search engines that share similar search equation writing rules. We adapted the string for Ovid, notably for double wildcards in the same item and for left truncation, that are not supported. We adapted the writing for Google Scholar in line with the total number of characters (256) allowed by the search engine [6]. Our search string was built through a step-by-step process.

#### 4.2.3 Testing for performance of the search

At each refining step of the search string, we tested the relevance of the search outcomes with the following indicators:

- Number of records: Using our final search string, we obtained 6682 records cumulated over the four search engines.
- Hit rate: i.e. the percentage of relevant articles within a pool of 100 randomly picked records; we aimed at maximising it.
- Miss rate: i.e. the percentage of references belonging to the test-list that were not retrieved by the search string. To calculate the miss rate, we established a test-list prior to conducting the searches. Our test-list included 39 meta-analyses presenting impacts of an agricultural management on a biodiversity outcome, relevant for our research question; the test-list was established by the authors of the study and the stakeholders of the project. We aimed at minimising the miss rate. Using our final search string, 37 over 39 references were retrieved within the 6682 records i.e. representing a miss rate of 8%.

These three indicators were scrutinised at each refining of the search string, in order to find a compromise allowing us to run the next steps of the protocol within the time constraints of the project.

#### 4.2.4 Publication databases searched, search engines, and supplementary searches

The following four multidisciplinary databases and search engines were used for the literature search:

1. **Web of Science (WoS) Core Collection** (producer: Clarivate Analytics, USA), coverage is from the year 1900 to the present day. Multidisciplinary, peer-reviewed. url: https://www.webofscience.com/wos/
2. **Scopus** (producer: Elsevier, The Netherlands). Coverage is from the year 1800 to the present day. Broad, multidisciplinary peer reviewed. url: https://www.scopus.com/search/form.uri
3. **Ovid** (producer: Wolters Kluwer group, USA), coverage is from the year 1946 to the present day. Broad, multidisciplinary, peer reviewed. url: https://ovidsp.ovid.com/
  Ovid aggregated results from two databases: (i) Cab Abstracts (producer: CABI), emphasising international agricultural literature, and (ii) Agricola (producer: Department of Agriculture, USA), indexing a wide variety of agricultural and allied fields literature.
4. **Google Scholar** (producer: Google, USA). Broad, multidisciplinary peer reviewed and grey literature. We focused on the first 400 search results, thus doubling the threshold suggested by Haddaway et al. [6] organised by relevance. url: https://scholar.google.com/
  Google Scholar search was performed using the free Publish or Perish software (producer: Anne-Wil Harzing, www.harzing.com).

We performed WoS, Scopus and Ovid searches through our CIRAD institutional access. We completed additional bibliographical searches as following:

5. We checked the references of included meta-analyses to identify any new relevant meta-analyses. We also checked existing reviews of meta-analyses published in agronomy [7,8].
6. We considered other literature sources identified as relevant by the stakeholders in order to gather grey literature or literature sources that would not have been reached otherwise.

#### 4.2.5 Managing duplicates

To identify duplicates between publication sources, we used the function *find_duplicates* (arguments: match variable “title”, match function “stringdist”, method “lv”) in the *revtools* R package [9]. This function scans the closely-written records titles and produces a variable that we added in the records table in a new column. We used R for Windows v. 4.1.0 (R Core Team, 2021) and RStudio Desktop v. 1.4.1717 (RStudio Team, 2021). We then manually verified the records table to validate (or not) the flagged duplicates by *revtools*.

### 4.3 Article screening process and study eligibility criteria

#### 4.3.1 Study inclusion and exclusion criteria

To be retained, screened articles had to meet the eligibility criteria listed in Table 3.

**Table 3:** Eligibility criteria, arranged by PICOC components.

PICOC component	Definition
Eligible Population(s)	Any non-crop biodiversity (associated biodiversity), sampled or observed.
Eligible Intervention(s)	The article provides a clear description of the agricultural management practices to be tested in the ‘treatment’ plots (cf. table 2).
Eligible Comparator(s)	The article provides a clear description of the ‘control’ plots that were used as baseline for the mean effect to be presented. These controls can be for example under “conventional” agriculture practices or natural habitats. The soil characteristics, dates, and methods of sampling/observation must be comparable between ‘treatment’ and ‘control’ plots.
Eligible Outcome(s)	The article must provide quantitative data on the effects of interventions and comparators on biodiversity. The provided data must allow us to extract the mean effect size and dispersion of effect sizes (e.g., confidence interval) and the sample size. Biodiversity outcomes may include (i) taxonomic metrics (based on richness, abundances, evenness…) and/or (ii) functional trait-based metrics, and/or (iii) activity metrics, and/or (iv) phylogenetic diversity metrics. The metrics describe the alpha (sampled field/crop scale) biodiversity.
Eligible Context	The article must include a first-order meta-analysis investigating at least one agricultural management as intervention and its effects on at least one biodiversity outcome in croplands (i.e. grasslands-only effect sizes are excluded). Meta-regressions are not retained.

**Fig. 3:**
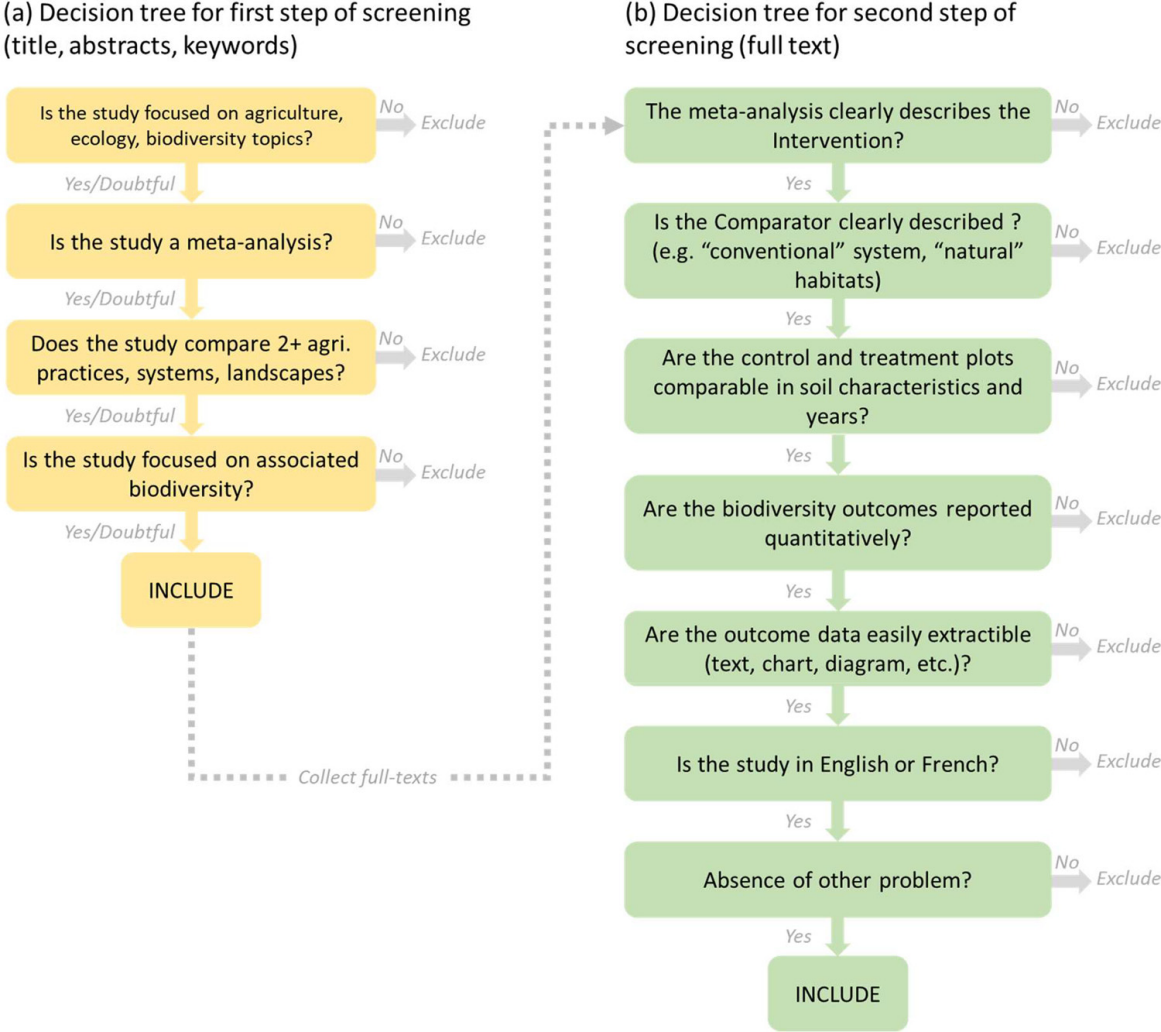
Decision trees for the first (a) and second (b) step of the screening procedure.

Based on our inclusion and exclusion criteria we designed two decision trees, one for each step of the screening procedure (see Fig. 3). Each retrieved article was thus assessed for relevance using the eligibility criteria and decision trees. No study design types were excluded during the screening stages. This was done in order to achieve a comprehensive evidence-base.

#### 4.3.2 Article screening and consistency checking

In accordance with the pre-defined screening and article eligibility criteria (detailed in §4.3.1), article selection followed a two-step screening process. During the first step, titles and abstracts of all retrieved references were screened. This step was performed using the freely accessible online tool Abstrackr (http://abstrackr.cebm.brown.edu/). During the second step, full texts were screened.

At each step, we retained articles meeting the inclusion criteria. During the title and abstract screening process, in case of doubt of the presence of an inclusion criterion (or if information was absent) the article in question was tagged as “doubtful” and checked at the full-text screening stage.

In order to check that consistent and repeatable decisions were made i.e. to minimise the risk of false negatives (articles containing relevant information incorrectly rejected during screening), adherence to the eligibility criteria was assessed between the two reviewers using a Cohen's Kappa test, at the start of each screening stage. Two benchmark runs and discussions about inconsistencies resulted in a satisfying 0.8 Kappa score which represented a substantial agreement [10].

**Fig. 4:**
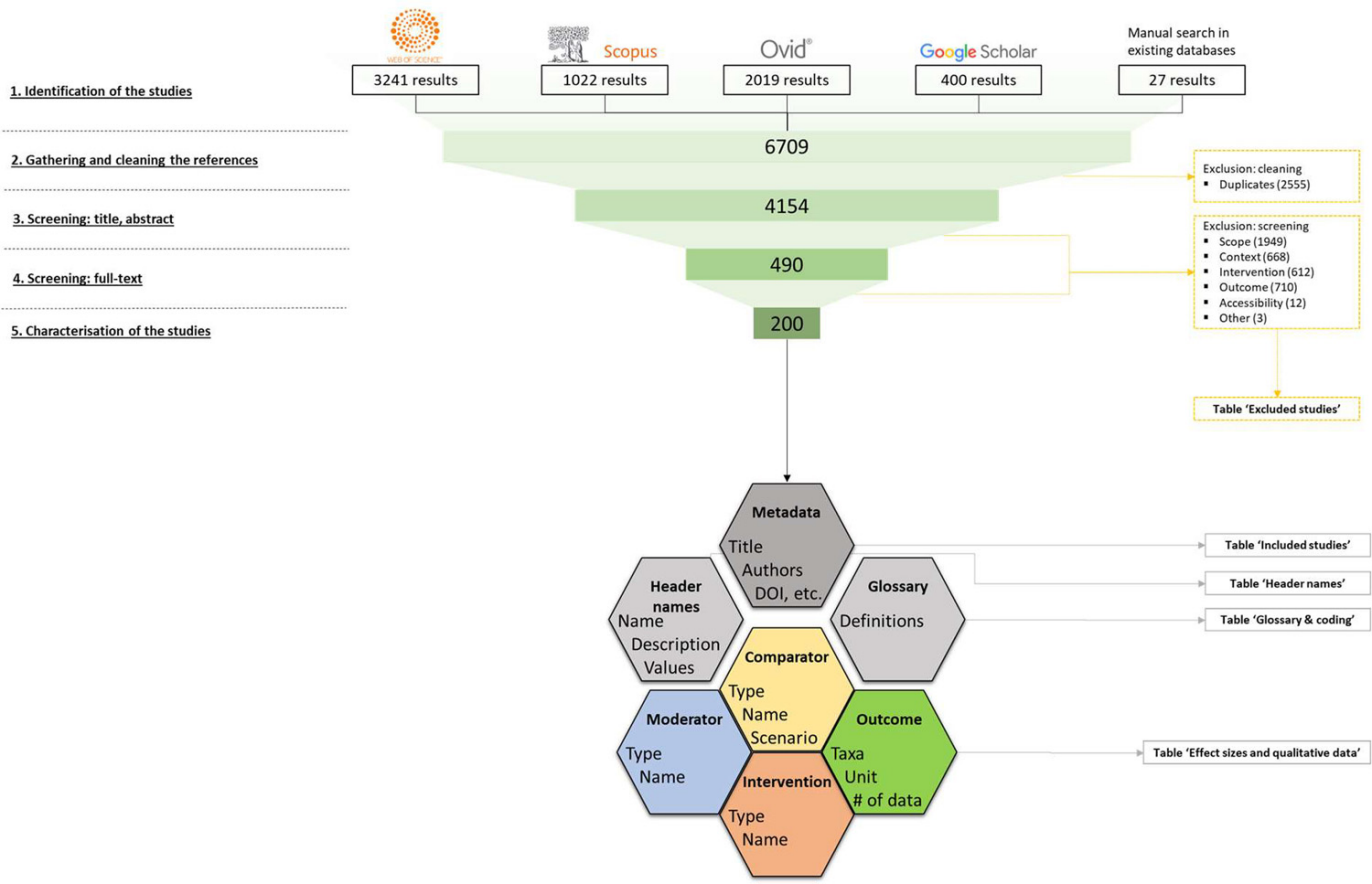
Flow diagram reporting the different steps of our methodology and the number of relevant literature references retained at each stage.

We present the whole process of search engines requests, duplicates identification, articles screening and inclusion under the form of a PRISMA flow diagram [11] in Fig. 4.

### 4.4 Data extraction & coding

**Fig. 5:**
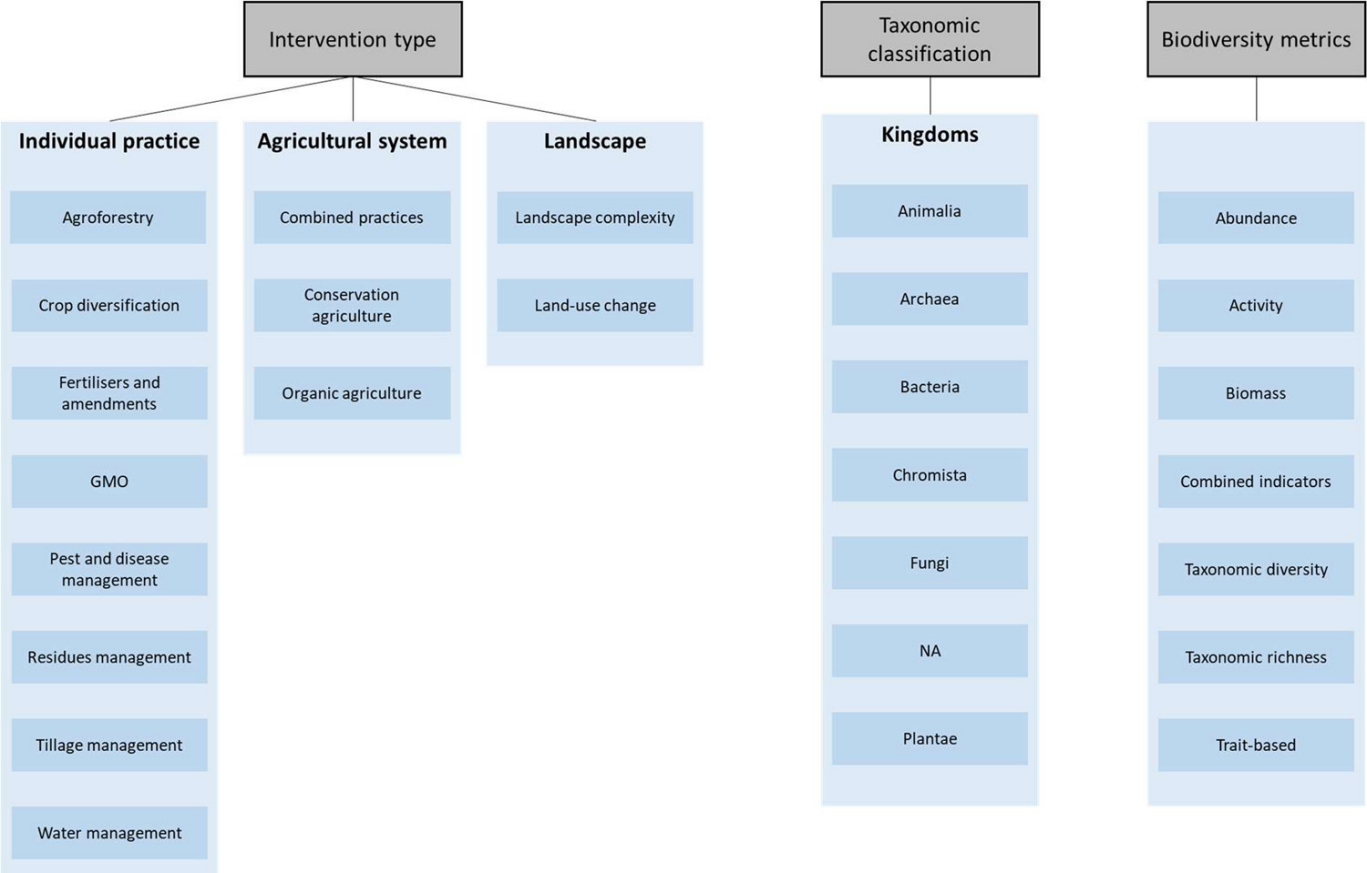
Ontology listing the entries available in our database for the type of intervention and the biodiversity outcome (taxonomic group, biodiversity metric). GMO: genetically modified organism.

The first step of data extraction consisted in extracting the metadata (title, authors, date…) of the articles and was carried out alongside reference extraction and management during the screening process. This step allowed us to fill the table “Included studies” and “Excluded studies”. The second step consisted in extracting - within each included article - each effect size representing a relevant Intervention*Outcome combination, which allowed us to fill the table “Effect size and qualitative data”. Each effect size was qualified and quantified with data describing notably the intervention, the comparator, the moderators, the biodiversity outcome (taxon and metric). Moderators are qualitative or quantitative information on cofactors that may influence the Outcome; they may concern for example the geographical extent of the meta-analysis, the climate concerned by the effect size, the crop species, the soil parameters, etc. We present the principal entries available in our database for the intervention type, the taxonomic group and the biodiversity metric, for each effect size, in Fig. 5.

Each of the meta-analysis presents mean effect sizes but also factors that may influence the value of these effect sizes’ Outcome. In the case of meta-analyses, such factors are called Moderators. In our dataset, we noted and provided the qualitative characteristics of each mean effect size and we extracted all moderators presented in each meta-analysis. Thus, Moderators that are present in our dataset are the ones presented by the authors of each meta-analysis. When coding our database, we harmonized the wording among Moderators and grouped them by topic for clarity.

When subgroup analyses were presented by a meta-analysis, we avoided extraction of redundant effect sizes by extracting in priority: effect sizes with the most detailed biodiversity information (e.g. taxon), then if detailed taxonomic information was not present we looked for ecological groups *sensu lato* (this may include functional groups, trophic guilds, etc.), then we looked for effect sizes with the most detailed intervention information. When subgroup analyses resulted in wrapping together several moderators and/or several levels of one moderator, these are mentioned as ‘Moderators_confounding’.

Example: In a meta-analysis, authors may present their mean effect sizes in different sub-group analyses showing: (1) the effects of 1 agricultural practice on *n* taxa regardless of the geographical extent, (2) the effects of 1 agricultural practice on biodiversity (all taxa grouped together) in *n* geographical extents (e.g. continents), and (3) the effects of 1 agricultural practice on the *n* taxa vs. *n* continents interactions.

Case (a): they provided only sub-groups (1) and (2). In that case, we prefer and extract the case (1) in which the geographical origin is coded as a confounding Moderator.

Case (b): they provided sub-groups (1), (2) and (3). In that case, we prefer and extract only the case (3) in which the geographical origin is coded as a Moderator with different levels (e.g. Europe, Africa, Asia, etc.).

Meta-analyses synthetise the mean effect of an Intervention compared to a Comparator on an Outcome. The choice of Intervention and Comparator can be different depending on the meta-analysis considered, and sometimes may differ from other meta-analyses within the same scientific topic. For example, one may synthetise the effects of No tillage versus Conventional tillage (case A), or the effects of Conventional tillage versus No tillage (case B). In our work we aim to compare results from different meta-analyses, thus, within the same topic, we have to homogenize Interventions and Comparators. When we had to invert the effects of a treatment on a control for that purpose, we: (i) coded a YES in the “Inverted_votecount” column, (ii) used the appropriate wording in the associated column “Scenario_comp”. Following our previous example, both meta-analyses (A and B) would fit in the same “No tillage vs Conventional tillage” scenario, while they are coded NA (case A) and YES (case B) in “Inverted_votecount”.

The coding strategy (ontology, vocabulary) of the Interventions and Outcomes is presented in the “Glossary and coding” sheet. The taxonomic information concerning Kingdom, Phylum (and/or subphylum), Class (or Subclass) and Order (or Superorder) was manually attributed to each effect size following the Catalogue of Life classification [12]. The agricultural management practices information classification was built after [13,14].

## Ethics Statements

The authors have read and follow the ethical requirements for publication in Data in Brief and confirm that the current work does not involve human subjects, animal experiments, or any data collected from social media platforms.

## CRediT authorship contribution statement

**Jonathan Bonfanti:** Conceptualization, Methodology, Investigation, Data curation, Writing – original draft. **Joseph Langridge:** Validation, Writing – review & editing. **Damien Beillouin:** Conceptualization, Methodology, Investigation, Data curation, Writing – review & editing, Supervision, Funding acquisition.

## Data Availability

A global database to quantify the impacts of agricultural management practices on terrestrial biodiversity (Original data) (Dataverse)
